# Characteristics of patients with newly diagnosed hematological malignancies referred for echocardiography

**DOI:** 10.3389/fonc.2024.1283831

**Published:** 2024-01-24

**Authors:** Jarosław Kępski, Sebastian Szmit, Ewa Lech-Marańda

**Affiliations:** ^1^ Department of Cardio-Oncology, Centre of Postgraduate Medical Education, Warsaw, Poland; ^2^ Department of Hematology, Institute of Hematology and Transfusion Medicine, Warsaw, Poland

**Keywords:** cardiooncology, echocadiography, hematology malignance, diastolic dysfunction, heart failure

## Abstract

**Objective:**

The importance of cardio-hemato-oncology programs is increasing. The main aim of the study was to identify all coexisting cardiovascular disorders in patients with new hematological malignancies referred for echocardiography during baseline evaluation before anticancer therapy.

**Material and methods:**

The study was based on 900 echocardiographic examinations performed within 12 months at the Institute of Hematology and Transfusion Medicine in Poland: 669 tests (74.3%) were dedicated to hemato-oncology patients at the different stages of cancer therapy, however almost a third of the tests (277, 30.8%) were part of a baseline evaluation before starting first line anticancer therapy due to newly diagnosed hematological malignancies.

**Results:**

The group of 277 patients with new hematological malignancies (138 women, 49.82%) with a median age of 66 years (interquartile range: 53-72 years) was included in the main analyses. The three most frequent new histopathological diagnoses were: non-Hodgkin lymphoma (63 cases; 22.74%), acute myeloid leukaemia (47 cases; 16.97%), and multiple myeloma (45 cases; 16.25%). The three most common clinical cardiology disorders were arterial hypertension (in 133 patients, 48.01%), arrhythmias (48 patients, 17.33%), and heart failure (39 patients, 14.08%). Among 48 patients with arrhythmias there were 22 cases with atrial fibrillation. The most frequently detected echocardiographic abnormality was Left Atrial Volume Index >34 ml/m2 which was present in 108 of 277 patients (38.99%) and associated with a significantly greater chance of concomitant diagnosis of arrhythmias (OR=1.98; p=0.048) especially atrial fibrillation (OR=3.39; p=0.025). The second most common echocardiographic finding was diastolic dysfunction 2nd or 3rd degree revealed in 43 patients (15.52%) and associated with a greater chance of simultaneous diagnosis of heart failure (OR=8.32; p<0.0001) or arrhythmias (OR=4.44; p<0.0001) including atrial fibrillation (OR=5.40; p=0.0003).

**Conclusions:**

In patients with newly diagnosed hematological malignancies left ventricular diastolic dysfunction is a common abnormality in echocardiography and may determine diagnoses of heart failure or arrhythmias.

## Introduction

Cardio-oncology is a dynamically developing scientific field. Its primary task is to provide appropriate quality of cardiological care for patients undergoing or after cancer therapy (1). The overriding goal is to ensure optimal anticancer treatment for patients with coexisting cardiovascular diseases diagnosed before or during cancer treatment. In many countries, scientific institutions create clinical programs dedicated to cardio-oncology (2). The emergence of cardio-oncology clinics is becoming a reality (3–5).

The development of modern therapies used in onco-hematology results in improved prognoses for patients. However, this favorable trend is limited by the increasing number of early and late cardiovascular complications mainly due to using novel anticancer drugs (6). They may reduce the overall survival rate and affect patients’ quality of life. For these reasons, programs dedicated to cardio-hemato-oncology have also been created (7, 8).

Cardiovascular risk stratification should be an integral element of each cancer patient assessment before initiating potentially cardiotoxic oncological therapy (9). Echocardiography seems to be necessary during such evaluation especially in hemato-oncology (10) Patients with a history of cardiovascular disease appear to be at greater risk of an unfavorable prognosis (11). The direct impact of cancer itself on the cardiovascular system is constantly underestimated (12).

The study aimed to summarize the first year of experience from the work of a new team of cardiologists at the Institute of Hematology and Transfusion Medicine in Warsaw, i.e. the Polish reference center for hematology. The main goal was to draw special attention to the importance of baseline cardiology evaluation of patients with a new diagnosis of hematological malignancies.

## Materials and methods

Indications for echocardiographic examinations of the heart performed from April 2021 to March 2022 were retrospectively analyzed. This period constituted the first 12 months of work of the new team of cardiologists at the Institute of Hematology and Transfusion Medicine in Warsaw, who are also members of the International Cardio-Oncology Society and certified cardio-oncologists.

**Figure 1:**
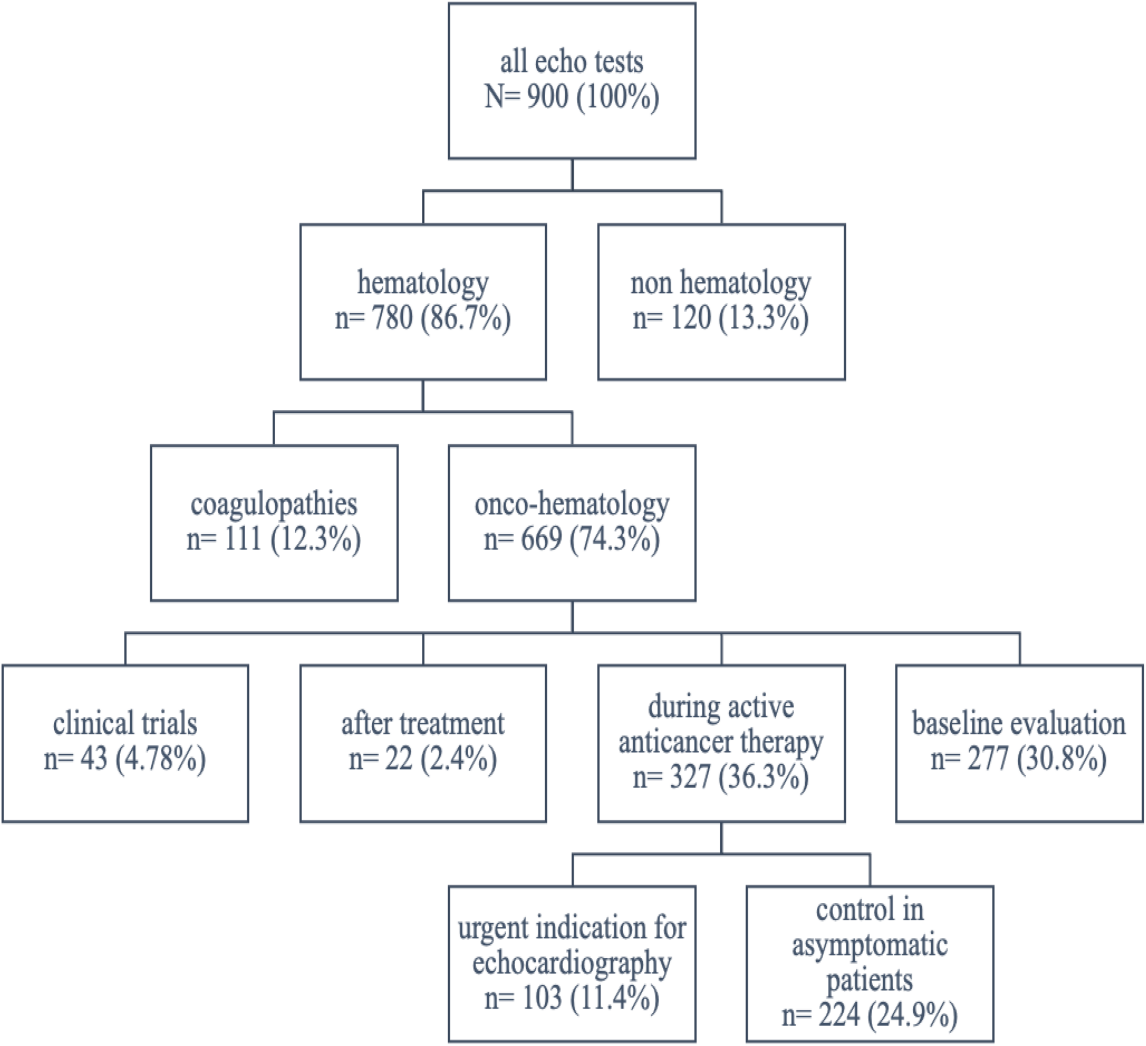
The flowchart of patients undergoing echocardiography.

There were 900 echocardiographic examinations performed for different clinical indications (**Figure 1**). Nearly one fourth of the examinations was performed in patients treated for reasons other than hematological malignancies: non hematology therapy (13.3%) or coagulopaties (12.3%). Most of the tests (669; 74.3%) were dedicated directly to hemato-oncology. The distribution of echocardiographic imaging in hemato-oncology was as follows:

- 277 (30.8%) patients at baseline evaluation
- 327 (36.3%) patients during active anticancer therapy
  ➣ 103 (11.4%) patients with new symptomatic events
  ➣ 224 (24.9%) patients under monitoring at different stages of hematological malignancy treatment:
    ○ 93 before hematopoietic stem cell transplantation (HSCT),
    ○ 51 with malignancy progression,
    ○ 80 as asymptomatic patients under surveillance.
- 22 (2.4%) patients during long-term follow-up after anticancer therapy
- 43 (4.78%) patients in clinical trials on an experimental therapy.

The final step was to identify coexisting cardiovascular diseases before anticancer therapy among patients starting treatment (baseline evaluation) and analyzing their echocardiographic diagnoses.

Echocardiographic studies were conducted on the patients in the left lateral decubitus position using a medical ultrasound device (EPIQ 5, Philips Medical System, USA). Left and right ventricular systolic function, left ventricular diastolic function, and exponents of pulmonary hypertension were assessed.

Echocardiograms were performed following the American Society of Echocardiography guidelines, using imaging in the following projections: substernal, parasternal in the long and short axis, apical four-, two- and three-chamber projections and suprasternal projection (13, 14). Ejection fraction was assessed using the Simpson method in the four- and two-chamber views. Diastolic function was assessed according to the American Society of Echocardiography and the European Association of Cardiovascular Imaging Guidelines (15). Not all tests were performed with simultaneous electrocardiogram monitoring. When assessing the diastolic function of the left ventricle, the average value of e’ from the septal and lateral values of the left ventricle was used. In our daily practice, we use TDI imaging of the right ventricle. Patients have their S’ value, i.e. systolic excursion velocity, assessed.

All cardiovascular disorders were diagnosed according to the thematically relevant recommendations of the European Society of Cardiology in force in Poland (16–18).

All clinical and echocardiographic diagnoses as nominal variables were expressed as percentages. Values of odds ratios (ORs) and respective 95% confidence intervals (CIs) were calculated by the logistic regression model to find relationships between echocardiographic findings and classical risk factors like arterial hypertension and coronary artery disease. The second purpose was to evaluate the chance of diagnosing heart failure or arrhythmias depending on different echocardiographic abnormalities, not only decreased ejection fraction (EF). All analyses were performed by STATISTICA software. The value of the test’s probability level p< 0.05 was understood as significant.

The study was approved by the Bioethical Committee of the Centre of Postgraduate Medical Education: opinion number: 74/2022, Warsaw 08.JUNE.2022.

## Results

The main analysis of the study was focused on 277 patients (138 women, 49.82%) with a median age of 66 years (interquartile range: 53-72 years) who had newly recognized hematological malignancies and had undergone echocardiography as an element of risk stratification before anticancer therapy. The frequency distribution of new blood cancers diagnoses is presented in **Table 1**.

**Table 1:** Eight most frequent histopatological diagnoses of new hematological malignancies among 277 patients referred to echocardiography.

	Diagnosis	Number of patients
1	Non-Hodgkin lymphoma	63 (22.74%)
2	Acute myeloid leukaemia (AML)	47 (16.97%)
3	Multiple myeloma (MM)	45 (16.25%)
4	B-cell chronic lymphocytic leukaemia (CLL)	33 (11.91%)
5	Myeloproliferative neoplasms BCR/ABL negative	25 (9.03%)
6	Acute lymphoblastic leukaemia (ALL)	13 (4.69%)
7	Hodgkin lymphoma	13 (4.69%)
8	Myelodysplastic syndromes	9 (3.25%)
	Total	248 (89.53%)

Among 277 patients with new hematological malignancies, three concomitant clinical cardiology diagnoses were the most common (**Table 2**): arterial hypertension (133 patients, 48.01%), arrhythmias (48 patients, 17.33%), heart failure (39 patients, 14.08%). The most common five new echocardiographic findings included (**Table 3**): LAVI >34 ml/m2 (108 cases, 38.99%), diastolic dysfunction 2nd or 3rd degree (43 cases, 15.52%), TRV >2.8 m/sec (40 cases, 14.44%), E/e’ >14 (18 cases, 6.55), left ventricular systolic dysfunction with ejection fraction EF<50% (10 cases, 3.61%).

**Table 2:** Clinical cardiovascular diseases identified in 277 patients with new hematological malignancies.

	Diagnosis	Number of patients (%)
1	Arterial hypertension	133 (48.01%)
2	Arrhythmias	48 (17.33%)
3	Atrial fibrillation (AF)	22 (7.94%)
4	Heart failure (HF)	39 (14.08%)
5	Coronary artery disease (CAD)	22 (7.94%)
6	Venous thromboembolic event (VTE)	10 (3.61%)

**Table 3:** Echocardiographic diagnoses in 277 patients with new hematological malignancies.

	Diagnosis	Number of patients (%)
1	Left ventricular systolic dysfunction with ejection fraction EF<50%	10 (3.61%)
2	Diastolic dysfunction 2nd or 3rd degree	43 (15.52%)
3	Left Atrial Volume Index: LAVI >34 ml/m2	108 (38.99%)
4	E/e’ >14	18 (6.50%)
5	Tricuspid regurgitation velocity: TRV >2.8 m/s	40 (14.44%)
6	Tricuspid annular plane systolic excursion: TAPSE <17 mm	6 (2.17%)
7.	Peak S wave velocity of the lateral tricuspid annulus by tissue Doppler imaging: RVS’<9.5 cm/s	8 (2.89%)

Patients with arterial hypertension or coronary artery disease had significantly greater chance of diagnosis of diastolic dysfunction grade 2nd or 3rd (OR=4.23 or OR=3.46 respectively, p<0.05 for both). Diagnosis of EF<50% was significantly associated with coronary artery disease (OR=9.19; p=0.001) and borderline related to arterial hypertension (**Table 4**).

**Table 4:** The odds ratio of recognition of five main echocardiographic abnormalities in relation to arterial hypertension and coronary artery disease.

	LAVI >34 ml/m2	2nd or 3rd degree diastolic dysfunction	TRV >2.8 m/s	E/e’ >14	EF <50%
Arterial hypertension	OR=3.52 95%CI:2.05-6.05 p<0.0001	OR=4.23 95%CI:1.98-9.04 p=0.0002	OR=2.39 95%CI:1.17-4.9 p=0.02	OR=9.66 95%CI:2.16-43.14 p=0.003	OR=4.51 95%CI:0.93-21.8 p=0.06
Coronary artery disease	OR=4.31 95%CI: 1.52-12.27 p=0.006	OR=3.46 95%CI:1.35-8.89 p=0.01	OR=2.8 95%CI:1.07-7.42 p=0.04	OR=1.56 95%CI:0.33-7.34 p=0.57	OR=9.19 95%CI:2.36-35.74 p=0.001

Diastolic dysfunction 2nd or 3rd degree on echocardiography was strongly related to a greater chance of diagnosis of heart failure (OR=8.32; p<0.0001) or arrhythmias (OR=4.44; p<0.0001) including atrial fibrillation (OR=5.40; p=0.0003) (**Table 5**).

**Table 5:** The odds ratio of diagnosing heart failure or arrhythmias (including atrial fibrillation) in relation to echocardiographic abnormalities.

	Heart failure	Arrhythmias	Atrial fibrillation
LAVI >34 ml/m2	OR=1.53 95%CI: 0.72-3.29 p=0.27	OR=1.98 95%CI: 1.00-3.92 p=0.048	OR=3.39 95%CI: 1.16-9.90 p=0.025
2^nd^ or 3^rd^ degree diastolic dysfunction	OR=8.32 95%CI: 3.82-18.1 p<0.0001	OR=4.44 95%CI: 2.14-9.20 p<0.0001	OR=5.40 95%CI: 2.15-13.56 p=0.0003
TRV >2.8 m/sec	OR=2.85 95%CI: 1.26-6.44 p=0.01	OR=1.93 95%CI: 0.88-4.24 p=0.1	OR=1.65 95%CI: 0.57-4.78 p=0.36
E/e’ >14	OR=7.83 95%CI: 2.86-21.41 p<0.0001	OR=7.4 95%CI: 2.73-20.06 p<0.0001	OR=7.5 95%CI: 2.48-22.71 p=0.0003
EF <50%	#	OR=5.19 95%CI: 1.43-18.79 p=0.01	OR=3.08 95%CI: 0.61-15.57 p=0.17

#all patients with EF<50% had heart failure diagnosis.

There were other interesting relationships between echocardiographic findings and clinical cardiology diagnoses (**Table 5**):

- Left Atrial Volume Index LAVI >34 ml/m2 was found significantly more often together with a diagnosis of arrhythmias (OR=1.98; p=0.048) especially with atrial fibrillation (OR=3.39; p=0.025). LAVI >34 ml/m2 was not significantly associated with a chance of diagnosis of heart failure.
- Tricuspid regurgitation velocity TRV >2.8 m/sec could indicate a higher chance of a diagnosis of heart failure (OR=2.85; p=0.01) but not arrhythmias.
- E/e’ >14 was strongly associated with a greater chance of concomitant diagnosis of heart failure (OR=7.83; p<0.0001) or arrhythmias (OR=7.4; p<0.0001) including arterial fibrillation (OR=7.5; p=0.0003).

All 10 patients with recognized EF < 50% had concomitant diagnoses of heart failure.

Among 43 patients with new diagnoses of hematological malignancies and recognized diastolic dysfunction 2nd or 3rd degree: 10 had coexisting atrial fibrillation (p<0.0001) and 8 had concomitant LVEF<50% (p<0.0001). It should be highlighted that only 2 patients had simultaneously atrial fibrillation, LVEF <50% and diastolic dysfunction 2nd or 3rd degree.

## Discussion

The new ESC guidelines dedicated to cardio-oncology recommend cardiological and echocardiographic assessment of cancer patients at various stages of anticancer treatment, i.e. as in our study (19). Echocardiography should be part of the baseline cardiovascular risk stratification before anticancer treatment. The coexistence of cardiac diseases may be associated with a worse prognosis in patients treated for cancer (20). Cancer and its treatment favor the development of new and progression of existing cardiac diseases. Therefore, risk stratification before anticancer therapy seems extremely important (21). To a large extent, in terms of many anticancer drugs, this stratification is based on the result of echocardiography, although clinical diagnoses are also of integral importance (9). It should be emphasized that the initial risk stratification has its prognostic justification (11, 22).

Our analysis confirms a high frequency of diagnoses of cardiac diseases among patients with new hematological malignancies. Our data show that 14.08% of patients with echocardiographic examinations at baseline had a diagnosis of heart failure, and 17.33% had arrhythmias. Echocardiographic findings were even more critical: 3.61% had left ventricular systolic dysfunction with EF <50%, 15.52% had grade 2 or 3 diastolic dysfunction, and 14.44% of patients had TRV >2.8 m/2. Notably, as many as 38.99% of patients had LAVI >34 ml/m2 and that was the most frequent echocardiographic abnormality in this population. In our opinion, both LAVI and diastolic function should be evaluated not only at baseline but also during active anticancer treatment, which seems necessary to monitor them. Such modern monitoring may explain some reasons for the diagnosis of new episodes of heart failure or arrhythmias related to anticancer drugs activity.

The ESC guidelines emphasize the need to perform planned examinations serially, even in asymptomatic patients, during their anticancer treatment. This course of treatment makes it possible to recognize echocardiographic signs of heart damage very early. As a result, it is possible to start secondary cardio protection effectively. Echocardiographic assessment of patients after completion of anticancer treatment is of critical importance as part of long-term follow-up including reassessment of the patient at the time of cancer recurrence. In hemato-oncology, this becomes particularly important at the moment of qualification for bone marrow transplantation (23).

Cardiovascular risk stratification in cardio-oncology is a dynamic process. A cancer patient with low or moderate risk may become a high- or very-high-risk patient during or after oncological treatment if new echocardiographic abnormalities occur. In this dynamic process of risk re-stratification, the basis is, of course, performing echocardiography before anticancer treatment.

Our analysis concerns the period before the announcement of the latest guidelines of the European Society of Cardiology, created in cooperation with the European Hematology Association. The aim was to illustrate the situation before the publication of the guidelines, which for the first time, defined the principles of cardiovascular risk stratification and formal echocardiographic assessment in oncology and hematology.

Echocardiography plays a vital role in cardio-oncology (24). The number of important echocardiographic diagnoses during routine scheduled echocardiographic examinations supports the necessity of performing these examinations regularly and frequently in everyday practice, as recommended by experts (10). This is the optimal way to plan early prevention (25). Our data show how important it is to assess cancer patients optimally at baseline and have additional echocardiographic parameters associated with the diastolic function of the left ventricle or function of the left atrium to compare them during active cancer therapy.

Optimal baseline evaluation of cancer patients may predict symptomatic and sometimes acute cardiac events as an element of cardio-oncology care (26). At baseline the most common diagnoses can be clinical problems related to arterial hypertension, heart failure and arrhythmias as in our study. Hematological patients with such baseline diagnoses are most vulnerable to further severe complications during active cancer therapy. These events have recently been discussed in a consensus published by the experts from the Acute CardioVascular Care Association (ACVC) and the ESC Council of Cardio-Oncology (27).

In onco-hematology, drug toxicity can affect any structure of the cardiovascular system. Therefore, cardio-hemato-oncology includes not only the diagnosis and treatment of heart failure but also of many vascular complications (28). Moreover, it has been confirmed that arterial hypertension together with coronary artery disease presents one of the most important risk factors in cardio-hemato-oncology (29). Arterial hypertension was the most common coexisting vascular problem in patients with newly diagnosed hematological malignancies in our study. Preexisting coronary artery disease was also recognized in some patients. Both arterial hypertension and coronary artery disease are well- known and important risk factors for the development of heart failure in general cardiology. Our h emato-oncology study highlights how both provoke greater chance of finding different echocardiographic abnormalities (**Table 4**).

In our observations, arrhythmias were the second most common clinical problem with atrial fibrillation comprising 7.94% of cases. This type of arrhythmia will undoubtedly focus the attention of cardio-oncologists on both the implementation of rhythm control strategies and antithrombotic prophylaxis (30). In the world of cardio-oncology growing attention is being paid to cardiac arrhythmias, mainly atrial fibrillation (31, 32). Cardiac arrhythmias, especially atrial fibrillation and other tachyarrhythmias, may indicate subclinical left ventricle dysfunction in hemato-oncology (33). The complex proarrhythmic mechanism in this group of patients results from water and electrolyte disorders, advanced age, coexisting cardiovascular diseases, and the direct effect of anticancer drugs (34).

Atrial fibrillation is becoming the special focus of arrhythmias in cardio-hematology (35, 36). Patients with previously diagnosed atrial fibrillation are at higher risk of death and cardiovascular complications in connection with stem cell transplantation (37). In patients receiving anthracyclines, a significant relationship has been revealed between heart failure development and atrial fibrillation recognized before or during chemotherapy (38). It seems extremely important that our study shows that even baseline echocardiographic parameters related to diastolic dysfunction may help predict a greater chance of the development of arrhythmias including arterial fibrillation.

In our analysis, diastolic dysfunction (2nd or 3rd degree) was the second most common abnormality recognized by echocardiography. This was a determining factor in many cases of diagnosed heart failure before anticancer therapy. Moreover, a diagnosis of diastolic dysfunction (2nd or 3rd degree) was more common than left ventricular systolic dysfunction with EF<50%. Other exponents of diastolic dysfunction like E/e’ > 14 or TRV >2.8 m/s were more frequent as well (**Table 5**). The importance of diastolic dysfunction is underestimated in cardio-oncology. Our study confirms that the assessment of diastolic function before oncological treatment is crucial and echocardiography should not be limited only to parameters of systolic cardiac function. This may help diagnose heart failure with preserved left ventricular ejection fraction and identify very high-risk patients in cardio-oncology.

Evaluation of left ventricular diastolic function is one of the elements of echocardiography. To identify it, we used: peak flow velocities through the mitral valve - waves E and A, the velocity of the tissue mitral annulus e’ in the Doppler examination, mean E/e’, the indexed volume of the left atrium and maximum velocity of the regurgitant wave through the tricuspid valve (15). The latest ESC guidelines do not recommend additional specific parameters for cancer patients. Meanwhile, diastolic dysfunction may precede the appearance of systolic dysfunction and full-blown heart failure at significantly lower doses of anthracyclines (39, 40). Such early diagnosis is possible only if all cancer patients are evaluated by means of echocardiography before cancer therapy. It should be highlighted that in patients with active cancer, deterioration of diastolic function may be a risk factor for all-cause mortality (41).

Our publication shows the validity of diastolic function assessment before anticancer treatment. Further careful observation will be required as to what drugs used in hemato-oncology affect the development of diastolic dysfunction of the left ventricle; hence it is important to assess the parameters of diastolic function in the initial examination. Another remaining issue is determining the prognostic role of diastolic dysfunction in hemato-oncology.

### The limitations of the study

The study is purely epidemiological in nature to reflect the scale of cardiac problems in hemato-oncology. The authors tried to prove that the permanent employment of cardiologists in a leading hematology center in the country has a deep justification. The study analyses the experience of cardiologists gained by them in the period of 12 months before the publication of the first European guidelines on cardio-oncology. At that time there was no uniform echocardiographic monitoring algorithm developed and the indications for testing asymptomatic patients depended on the hematologist’s decision taking place at important moments in the treatment of blood cancer, i.e. when the next line of treatment was planned. This situation changed after the publication of the ESC guidelines. The experience regarding monitoring of patients during active cancer therapy with a comparison period before and after the publication of ESC guidelines will be the topic of our subsequent study showing how much the diagnosis of cardiac events increases. Meanwhile, we have proven how important it is to perform echocardiography in all patients with new hematological cancers. The percentage of abnormalities in echocardiography is significant.

The next significant limitation is the lack of global longitudinal strain (GLS) value measurements. The reason is very simple, the evaluation of GLS became standard in our hematology center after the publication of ESC guidelines.

The last limitation is the fact that fluid overload and sinus tachycardia make measurements difficult in hemato-oncology. As confirmed by our study, common arrhythmias including atrial fibrillation will significantly complicate the assessment of left ventricular diastolic dysfunction. The diagnostic accuracy may be questionable. Moreover, it is still unknown in cardio-oncology if the occurrence of abnormal diastolic function parameters may reflect subclinical LV dysfunction. Such an understanding of modern cardio-oncology requires an evaluation of diastolic function in each patient before anticancer therapy.

## Conclusion

The new clinical diagnoses in hemato-oncology coexist mainly with arterial hypertension, heart failure and arrhythmias including atrial fibrillation.

Abnormalities in left ventricular diastolic function are clearly more frequent than systolic dysfunction in patients with newly diagnosed hematological malignancies and are associated significantly with concomitant diagnoses of heart failure or cardiac arrhythmias.

## Data availability statement

The raw data supporting the conclusions of this article will be made available by the authors, without undue reservation.

## Ethics statement

The studies involving humans were approved by Bioethical Committee at the Medical Center for Postgraduate Education in Warsaw. The studies were conducted in accordance with the local legislation and institutional requirements. The participants provided their written informed consent to participate in this study.

## Author contributions

JK: Conceptualization, Investigation, Writing – original draft, Writing – review & editing. SS: Conceptualization, Writing – original draft, Writing – review & editing. EL-M: Writing – review & editing.
